# An Agent-Based Epidemic Simulation of Social Behaviors Affecting HIV Transmission among Taiwanese Homosexuals

**DOI:** 10.1155/2015/867264

**Published:** 2015-03-01

**Authors:** Chung-Yuan Huang

**Affiliations:** Department of Computer Science and Information Engineering, School of Electrical and Computer Engineering, College of Engineering, Chang Gung University, 259 Wen Hwa 1st Road, Taoyuan 333, Taiwan

## Abstract

Computational simulations are currently used to identify epidemic dynamics, to test potential prevention and intervention strategies, and to study the effects of social behaviors on HIV transmission. The author describes an agent-based epidemic simulation model of a network of individuals who participate in high-risk sexual practices, using number of partners, condom usage, and relationship length to distinguish between high- and low-risk populations. Two new concepts—free links and fixed links—are used to indicate tendencies among individuals who either have large numbers of short-term partners or stay in long-term monogamous relationships. An attempt was made to reproduce epidemic curves of reported HIV cases among male homosexuals in Taiwan prior to using the agent-based model to determine the effects of various policies on epidemic dynamics. Results suggest that when suitable adjustments are made based on available social survey statistics, the model accurately simulates real-world behaviors on a large scale.

## 1. Introduction

**Figure 1:**
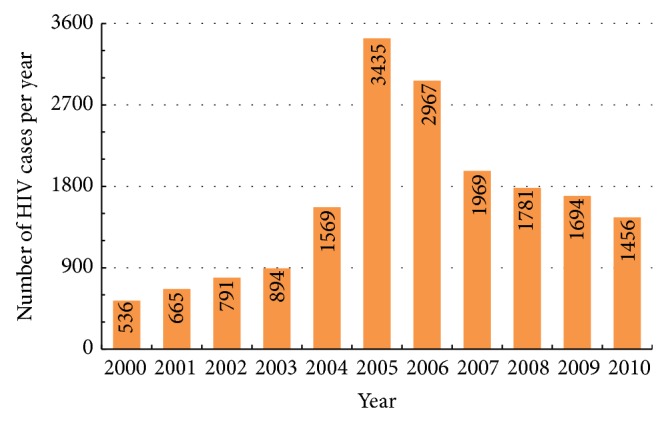
Taiwan HIV epidemic curves from 2000 to 2010.

According to the World Health organization (WHO) [1], more than 4.7 million people in Asian countries were HIV-positive at the end of 2008, with 350,000 newly infected individuals and 330,000 AIDS-related deaths in that year alone. While the 2008 AIDS-related mortality rate in South-East and South Asia was approximately 12% lower than the 2004 peak, the number of deaths in 2008 was still more than three times higher than that recorded in 2000. Between 2000 and 2009, the annual number of newly identified HIV-positive Taiwanese people increased from 536 to 1,694 (Figure 1). Since the peak number in 2005, the annual incidence rate between 2006 and 2010 declined by between 5% and 34% (average 14.9%). Homosexual activity (“men who have sex with men” or MSM) is believed to be the primary mode of HIV transmission in Taiwan today—57% of all cases recorded in 2008 alone [2]. Developing an HIV simulation model to understand HIV epidemic dynamics within male homosexual communities in Taiwan is therefore considered important for assessing the efficacies of HIV prevention efforts and intervention strategies associated with MSM activities [3, 4].

Some researchers have noted that the topological (connectivity) features of sexual networks exert considerable influences on HIV epidemic dynamics [5–9]. These features support analyses of the subtle details of HIV epidemic dynamics that population-based simulation approaches do not. Brailsford et al. [10], Paltiel et al. [11], and Peterson et al. [12] have applied specific complex network models to investigate HIV epidemic dynamics. Other researchers have addressed topological features and statistical distributions of sexual networks without focusing on individual social behaviors that affect HIV epidemic dynamics (see, e.g., [13–19]). They also show a tendency to overlook the flexibility offered by agent-based social simulation approaches.

An HIV epidemic simulation with the characteristics of power-law degree distribution and small-world phenomena for reproducing sexual networks was developed for this project to assess the efficacies of specific HIV prevention and intervention strategies in Taiwan associated with social behaviors. A specific emphasis in this paper will be MSM activity in saunas and bars in northern Taiwan, with agents in the model capable of modifying their behaviors to the degree that they affect all homosexuals residing in that part of Taiwan. Following the lead of previous sexual network studies [20–24], the proposed model focuses on scale-free properties depicting small-world phenomena. We found that, during one three-month period, the numbers of sex partners of highly active homosexual males in the cities of Taipei and Taichung followed a scale-free distribution on a log-log-axis. One conclusion of the present study is that by addressing specific individual behaviors and implementing rules derived from social survey statistics, global observations can be accurately identified by simulations without introducing unreasonably large numbers of societal rules, thus reflecting small-world properties that are inherent to scale-free networks. We also found that the clustering results can be used to support analyses of factors such as drug and condom usage.

## 2. Previous Sexual Network Studies

In terms of approach, epidemic simulations can be classified as top-down or bottom-up [25]. Top-down simulations start by specifying group or subpopulation characteristics and behaviors and modeling their relationships with other groups [26]. One problem is that mathematical equations in top-down epidemic simulations can quickly become so complex that analytical representations of multivariate and complex stochastic processes become exceptionally difficult to manage in terms of computation and stability. In contrast, bottom-up epidemic simulations are based on a combination of an agent-based model and an underlying social network, with nodes representing agents and edges representing contacts between them. Bottom-up epidemic simulations have been widely used to explore social macrolevel phenomena by specifying the microlevel characteristics and behaviors of individuals and their contacts in well-defined social networks over specific time periods [27–30]. The HIV epidemic simulation described in this paper is in the second category.

The history of social network studies by sociologists has produced numerous insights regarding specific social contacts [31]. Typical top-down epidemic simulations such as the compartmental models proposed by Kermack and McKendrick [32] assume random contacts among individuals, especially in scenarios involving the airborne transmission of infections. In numerical simulations of sexually transmitted infections (STIs), randomness is deemphasized when addressing sexual contact networks and mixing patterns within populations, both considered important concepts in modeling sexual contact within well-defined subgroups [33, 34]. Assortative sexual mixing implies concordance between sex partners in terms of factors such as age, location of residence, ethnicity, socioeconomic status, and sex partner acquisition rate [35]. In contrast, disassortative mixing, which implies discordance in sex partner characteristics, allows the spread of STIs from groups with high STI prevalence (e.g., commercial sex workers) to members of other groups [36].

Wylie and Jolly's [37] use of voluntarily given contact information and tracing via clinical contacts in Manitoba, Canada, is a rare example of a reconstructed real-world sexual contact network. A total of 4,544 STI-positive individuals were asked to identify their sexual partners—information that in most cases is very difficult to obtain. According to their data, sexual contact networks consist of numerous small clusters. An important task for any researcher is to determine cluster overlaps and to analyze how they support the spread of HIV. Note that since informants such as those in the Manitoba study are already infected, the data they offer are often viewed as suspect, influenced by subjective biases, and lacking in insightful detail [38].

Sexual networks are dynamic. Since connections evolve at different rates over time, concurrent relationships within networks must be considered [17]. Researchers have made many attempts to develop models that reflect various levels of fidelity among partners, as well as transmission routes among individuals in nonconcurrent relationships within specific time frames. More active individuals exert greater influence in terms of the spread of STIs within their networks. This stresses the importance of model precision in representing more sexually active subpopulations.

Some contemporary approaches to social network research are based on topological properties found in real-world human societies, including high degrees of local clustering and small average distances between nodes. In these models, STIs are passed between linked nodes that are few in number but high in variance. This architecture is closely associated with small-world networks [14, 15, 20–23], whose nodes have strong connections with their closest neighbors (thereby influencing local networks) and very few random links connecting distant locations. An interesting property of these networks is the dramatic decrease in average distance between random nodes due to the small number of long-distance links.

Complex networks with small-world characteristics can be classified according to the node degree statistical distribution *P*(*k*) [39]. Three network classes that have been identified so far are single-scale, broadscale, and scale-free; sexual networks belong to the scale-free category [14, 15, 20–23]. Most scale-free network nodes have few connections with their adjacent nodes, and only small numbers of nodes have large numbers of connections. A sexual network exhibiting power-law degree distribution properties serves as an example of how epidemics can spread quickly, with scope and speed determined by link distribution rules. A large number of nodes with multiple links significantly increase the speed of a spreading epidemic on a large scale, thus explaining why degree distribution is central to sexual network model construction. Accordingly, a major challenge in HIV simulation design and analysis is obtaining reliable data.

## 3. HIV in Taiwanese Homosexuals

According to statistics from the Taiwan Center of Disease Control (TCDC) [2] of the 6,850 known HIV-1-positive individuals living in Taiwan 2004, 488 were foreigners, 1,874 had developed AIDS, and the male : female ratio was just below 14 : 1. This represents a 15% annual increase between 2004 and 2008—well above the United Nations criteria for a “serious increase.” According to risk factor analysis results, 35.6% of the increase could be attributed to heterosexual sex, 45% to MSM activity involving either homosexuals or bisexuals, 6.2% to injecting drug use behaviors (primarily syringe-sharing), and 12% to “other.” In other words, sexual behaviors accounted for the large majority of HIV transmission routes in Taiwan during this time period.

Gay Taiwanese men frequently find partners in saunas, bars, and secluded parks. To investigate MSM-related HIV-1 infections, in 2003 and 2004, the AIDS Prevention and Research Center of National Yang-Ming University used anonymous questionnaires to collect data on sexual contacts among gay sauna customers [40]. As part of this project, the center distributed information on sexually transmitted diseases (STDs) to sauna businesses. Concerned about the fact that an insufficient number of patrons would feel motivated to join the study, the researchers also used a mix of purposive and snowball sampling to increase the number of participants. The primary goals of the two-year study were to offer anonymous HIV-1 antibody and syphilis tests and counseling and to investigate risk factors for specific STDs among gay sauna customers. The researchers also used epidemiological methods to determine HIV-1 subtypes and to analyze correlations between subtypes and risk factors. Data were also collected on demographics, sexual behavior self-cognition, and knowledge of risk factors associated with HIV-1 and syphilis among men taking part in MSM behaviors in saunas. Of the 1,101 men who participated, 1,000 turned in usable questionnaires. For 40% of the participants it was their first time to be tested for HIV-1. Chen et al.'s main conclusions were the following. (a) MSM activity is a high-risk category for HIV-1 and syphilis infections in Taiwan, and ongoing research is required to monitor seroprevalence rates and to investigate associated risk factors; (b) the rate of condom usage within Taiwan's male homosexual community is unacceptably low, thus calling for intensive education efforts; and (c) a positive correlation exists between nonmedical drug use and HIV-1 infections, especially when drugs are used prior to visits to gay saunas (see also [2, 41, 42]).

## 4. Simulation Model

In addition to saunas and bars, Chen et al.'s [40] questionnaire addressed structural and behavioral aspects of the male homosexual populations in Taipei and Taichung, Taiwan's two largest cities. Topics included frequencies of visiting high-risk places, frequencies of change in sexual partners, behaviors leading to new connections, relationship durations, condom usage, and attitudes regarding HIV testing. Demographic data were deemphasized—no effort was made to look for associations between HIV infection and age, marital status, education level, or social behavior, since the study goal was to determine risk for entire networks to reflect the HIV epidemic among male homosexuals in Taiwan.

During model construction, high- and low-risk subpopulations were distinguished according to number of partners, condom usage, and a “faithfulness factor” indicating long-term monogamy. The term free link was used to describe situations in which a pair of agents has multiple partners and fixed links to describe two agents in a long term, multiyear fixed relationship. Topological features were incorporated into the network to take advantage of small-world, scale-free, and other complex network model properties. This required distributing agent links in a manner that ensures power-law degree distribution properties over a period of many years [14, 15, 21–23].

**Figure 2:**
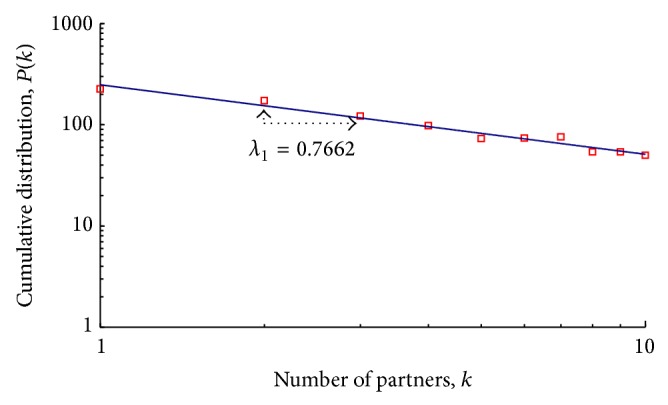
Scale-free distribution of sex partners among highly active homosexual males in Taipei/Taichung over three months (log-log axis). The low scaling exponent value (*λ* = 0.7662) indicates that individuals in this population tend to have many sexual encounters with different partners.

Sample data on cumulative numbers of sex partners during the preceding three months produced curves representative of a power-law pattern—specifically, with a low scaling exponent of 0.7662 for the number-of-partners distribution *k* ≥ 1 (Figure 2). Scale-free networks show a cumulative power-law distribution decay in the form *P*(*k*) ≈ *k* ^−*λ*_1_^, where *k* denotes the distribution of number of partners and *λ*_1_ the scaling exponent.

**Figure 3:**
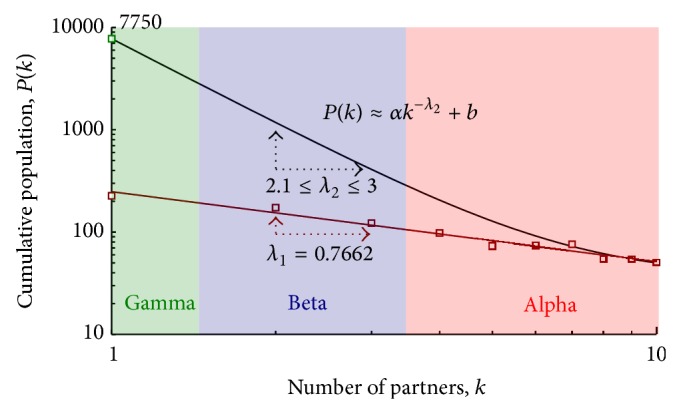
General distribution curves for the alpha, beta, and gamma subpopulations.

As stated above, the survey data only cover members of the high-risk subpopulation; the majority of male homosexuals in the two target cities might express a similar pattern, but with different scaling exponents and curve shapes. Since the goal was to achieve a power-law degree distribution among all simulation agents over a long time period, a *Pf* function or class of functions representing an accurate distribution of agent links was assumed. A plot of this function (generally expressed as *P*(*k*) ≈ *αk* ^−*λ*_2_^ + *b*) is shown in Figure 3.

Since individuals who have multiple sex partners are more likely to express different behaviors than those with only one partner, the curves can be divided into different sections representing subpopulations with unique profiles. The population in Figure 3 is divided into three clusters: alpha, in which a large number of sex partners are correlated with high-risk behaviors among a small number of individuals; beta, larger than alpha and characterized by a sexually active population expressing less extreme behaviors than alpha agents; and gamma, the largest population segment, representing the rest of the population. As suggested by the power-law pattern, each gamma individual has only one sex partner. The beta class was established because the gap between alpha and gamma patterns was considered too large to reflect real-world situations.

## 5. Design and Implementation of HIV Simulation Model

The proposed model consists of multiple layers, with each playing a role in describing and representing a specific function or phenomenon. Layer definition determines the roles of other layers in the simulation. Layers communicate with each other to exchange information regarding their computation results. Five layers were established for this study: agent, agent behavior, links, contact frequency, and epidemic. Layers can be categorized as belonging to an agent-based model consisting of agents and agent behavior layers, a social network model consisting of links and contact frequency layers, or an epidemic model. As explained above, a social network model has spatial, temporal, and intrinsic properties that evolve over time. A mix of global and individual behaviors was established to simulate agent influences on whole populations, but in a manner that reflects intrinsic societal changes via behavioral changes among a large number of individual agents. Societal global structure is defined in the agent layer, spatial concepts are reflected in the links layer, temporal concepts appear in the contact frequency layer, and intrinsic changes are modeled in the agent behavior layer. Suites of simple epidemiological progress statuses were added to the epidemic layer.

### 5.1. Agent Layer

The agent layer can be variously defined in terms of population structure, agent pool (Table 1), or individual agent characteristics (Table 2). In this study it represents three sexual activity patterns. The minority of extremely active agents (who play an important simulation role due to their high-risk behaviors) are found in the layer core. The second pattern consists of a number of sexually active agents that is far from a majority but exceeds the first pattern—that is, higher-than-average sexual activity, but lower risk behaviors in terms of condom usage and relationship duration. The third pattern, consisting of the large majority of relatively inactive individuals in a population, represents a pool with infection potential, but at a much lower level of risk.

### 5.2. Agent Behavior Layer

**Figure 4:**
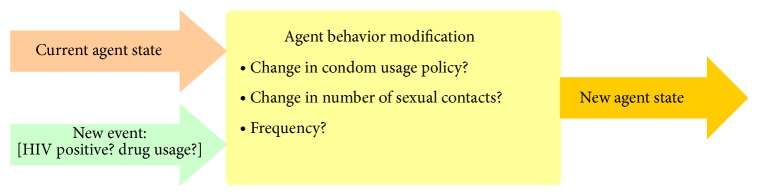
Layered agent behavior implementation.

This layer is defined as initial agent behavior and environmental adaptation (Figure 4). The first of two major behavioral changes that can be tested in an HIV epidemic simulation is an increase in condom usage—a parameter that serves as a moderating factor. However, according to the data used in this study, there is no indication of an overall increase in condom usage in Taiwan's gay community, despite research showing that condom usage generally increases when agents are made aware of their HIV-positive statuses [40–42]. We assumed that (a) fixed link couples in long-term monogamous relationships were much less likely to use condoms than free link agents and (b) free link agents would be more careful during sexual encounters and more likely to insist that their partners use condoms.

The second behavioral change incorporated into simulations was willingness to be tested for HIV. It is difficult to track changes in testing behaviors due to the lack of statistical data over the past 20 years. Still, this factor is important in terms of behavioral decisions made by HIV-positive agents who are aware of their status, including condom usage and encouraging their peers and partners to be tested (Figure 4).

### 5.3. Links Layer

This layer reflects the spatial aspect of agent connections and determines the importance of epidemic size. Since new links support new contacts, they are considered a key HIV propagation vector. As mentioned in an earlier section, the majority of individuals in the model are involved in fixed, long-term relationships that can be measured in terms of years. Distinctions between free and fixed link relationships are important because agents generally make behavioral decisions based on context—frequency of visiting high risk locations, frequency of change of sex partners, relationship duration, condom usage, attitudes toward HIV testing, and so on. These distinctions also reflect disease propagation outside of high-risk subpopulations. Even though low-risk populations mostly consist of long-term monogamous relationships, a small number of free links can act as significant vectors for propagating the HIV virus. Once the virus enters a gamma subpopulation, the spread of the disease may be slow, but the overall number of infected individuals can be large due to the population size. Precise knowledge of the gamma population size is unnecessary; it is assumed to be much larger than the alpha and beta populations. However, screening alpha and beta populations is very important because they represent the core of sexual activity.

Fixed links are distributed among agents in the same subpopulation, with most having at least one partner during each simulation. Free links are distributed within and across various subpopulations, with the number of potential links for any agent dependent upon the population in question: alpha agents have more free links than beta agents, and beta agents have more free links than gamma agents. According to Liljeros et al. [21–23], the number of sexual contacts for any individual follows a power-law curve. We therefore assumed that any free link distribution of contacts over the lifetime of a single agent in any population also reflects a power-law pattern.

Assortative and disassortative population patterns reflect mixes of free and fixed link agents. Statistically speaking, even if an alpha population is small, an alpha agent is more likely to have free links with other alpha agents, resulting in easily identifiable assortative patterns. The same is true within beta subpopulations and to a lesser degree between alpha and beta subpopulations. Gamma populations are most likely to reflect assortative patterns, although some disassortative patterns are bound to appear due to size, despite the low probability of any individual gamma agent having free links. Free links in any gamma subpopulation are statistically disassortative, meaning that gamma agents with free links are very likely to connect with alpha or beta agents with numerous free links.

### 5.4. Contact Frequency Layer

The contact frequency layer reflects the temporal aspect of links between agents. Sexual networks are considered dynamic in two ways. The first is the number of new contacts established by an agent, with existing links lost when new links are created. It is important to understand the underlying mechanism, since creating a link with a new agent can expose a large portion of the network to HIV infection. The second aspect concerns existing link activity. Since some links are more active than others, the number of contacts with certain agents will be much larger than with other agents. For example, a fixed link between two gamma agents will show more activity than a free link in the same population. Even in an alpha population, a free link may only be activated once or twice. A trade-off exists between the number of links an agent can maintain and link duration: the more links an agent has, the weaker they are and the shorter their lengths are. In contrast, maintaining long-term fixed links may block the establishment of new links.

### 5.5. Epidemic Layer

**Figure 5:**
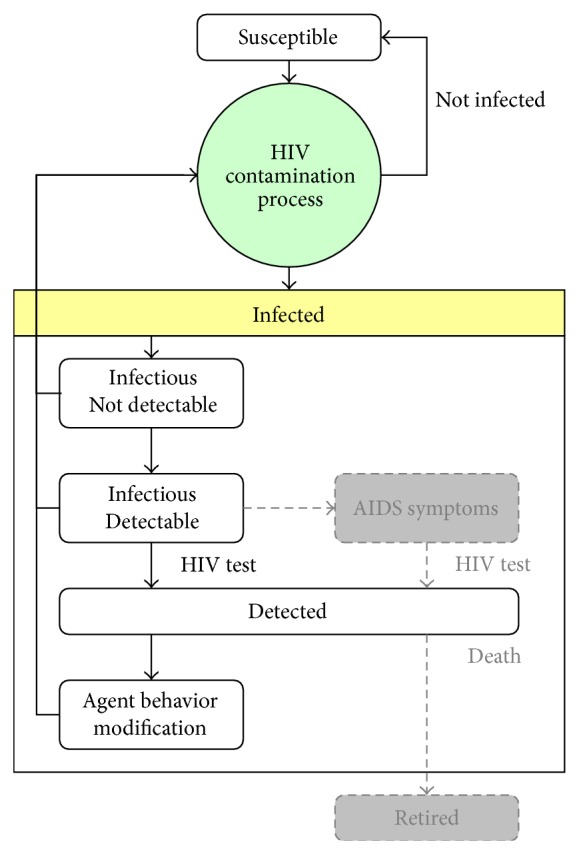
HIV and AIDS status development diagram. Our simulation is limited to HIV.

The study goal was to model growth in the number of HIV cases based on available data for a sexual contact network consisting of homosexual males. An epidemic model consisting of S (susceptible) and I (infected) individuals was used to simulate epidemiological progress resulting in status change, creating two infected subcategories in the process: AIDS and HIV status (Figure 5). AIDS individuals have the virus but do not yet have the antibodies required for detection. Lack of certainty about HIV status can influence behavioral decisions regarding sexual contacts and condom usage. Infected agents are automatically added to the HIV status subcategory after 6–12 weeks. Although HIV can be detected as early as two weeks following infection, we could not assume that all populations have access to state-of-the-art testing facilities.

All uncontaminated agents have an S status prior to virus exposure. Sexual contact between two S agents, with or without condoms, is insufficient for changing the status of either one; the same is true for sexual contact between two infected or HIV-positive agents, but not for contact between susceptible and infected or HIV-positive agents. If a susceptible agent uses a condom, it will reduce the probability of infection, but not by 100%. Furthermore, unprotected sex between susceptible and infected agents does not automatically result in new infections. As noted in Table 1, *p* denotes the probability of status change following sexual contact between susceptible and infected agents who use condoms and *q* denotes the probability of a susceptible agent being contaminated by an infected agent during unprotected anal intercourse.

### 5.6. System Implementation

**Figure 6:**
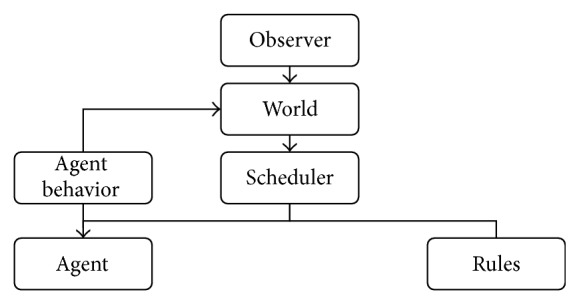
HIV epidemic simulation component diagram.

Implementation of the proposed five-layer simulation system entails communication between components to execute layer functions. A component diagram of the simulation framework is presented in Figure 6, and descriptions of the various components are given in Table 3. Agent layer and behavior are treated as two separate components, with links, contact frequencies, and epidemic layers managed according to the rules component, which includes a power-law shaping rule. The total time complexity of an experiment is O (*Population Size* ×* Max Contacts*).

We used the Borland C++ Builder's visual component library and event-driven programming model to develop the user interface and input/output functions of the simulation system. In addition to providing statistical reports and charts based on HIV epidemic data, the system lets users observe agent infection spreading scenarios in real time. Following compilation and conversion into an executable application, this system can be run on Windows using Dynamic Linked Library files.

## 6. Simulation Results

Due to screening limitations, the number of reported cases should not be viewed as an accurate representation of the actual number of cases, meaning that reported curves may not accurately reflect the evolution of the HIV epidemic over a period of years. However, we did assume that the spike in the number of reported cases in Taiwan during the time period covered by this project reflects an increased awareness and willingness to be tested and that a significant number of individuals who have unknowingly carried the HIV virus for a period of time are included in recent statistics. The primary hypothesis is that testing a larger percentage of the population will result in an increase in the number of reported cases. In terms of epidemic simulation, agents who test positive for HIV are more likely to be cautious about their sexual behavior, eventually slowing growth in the number of simulated cases and reducing the number of reported cases. This scenario must be considered when comparing reported and simulated case numbers.

Data from Chen et al. [40], Lai [41], and Lai et al. [42] were used to set the number of links, contact frequency, and drug and condom usage parameters for the alpha and beta populations. The parameters discussed in this section reflect important changes in social behaviors over the past 20 years. However, due to insufficient data on HIV infections among Taiwanese homosexuals for the same period, we had to use rough estimates for the evolution of changes in those behaviors. We did attempt to determine the plausibility of change in agent behavior based on feedback from an earlier generation of homosexuals. For example, we heard one comment that a growing number of young gay men are openly declaring their homosexuality, which may influence changes in alpha and beta populations. Another important change is the sharp increase in drug usage among young adults between the ages of 20 and 30, meaning that researchers must address the question of how drug usage affects safe sex practices and other behaviors. We will review models for estimating the effects of these new factors in the following sections.

### 6.1. Dynamic Change in the Population

In the proposed model, the overall population was divided into alpha, beta, and gamma subpopulations. The alpha and beta clusters increased in size based on the growing number of Taiwanese men openly declaring their homosexuality during the past 20 years; however, the lack of hard data on this trend makes it impossible to establish an accurate figure for simulation purposes. Since the proposed model is based on recently gathered information, such data are required in order to achieve a close fit between a simulation and the actual number of reported HIV cases.

**Figure 7:**
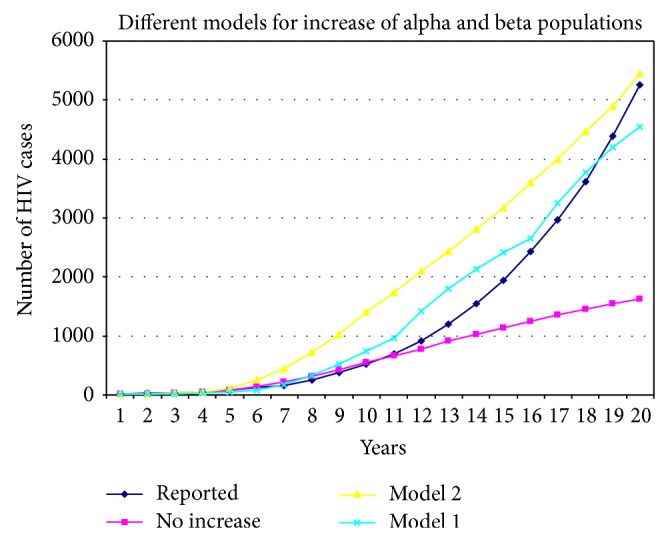
Results for two models of alpha and beta population increases.

In Figure 7, the curve labeled “No Increase” represents the number of simulated HIV cases without considering alpha and beta subpopulation increases. The curve clearly shows fewer infections than the actual situation—the slope flattens out over the long term, which is one result of saturation in the number of infected agents in the most sexually active subpopulations. One way to increase the number of infected cases would be to increase the number of exchanges between members of the alpha and beta subpopulations with members of the gamma subpopulation, but doing so would contradict the assumption of low levels of sexual activity for gamma individuals with anyone but their fixed partners. An alternative approach is to increase the number of alpha and beta agents.

Model 1 in Figure 7 reflects the potential for significant change in subpopulation size. While such changes are irregular, they can exert strong influences on individual behaviors—note the number of male homosexuals who “came out” in Western countries in the 1960s and 1970s. In model 1 we assumed three spikes in Taiwan during the past 20 years that resulted in significant increases in alpha and beta subpopulations and respective increases in the numbers of simulated HIV cases [43].

Model 2 reflects the assumption that growth in all three subpopulations is the result of linear increases over many years. While it is unlikely that this trend occurred in homosexual communities, the assumption does underscore the point that such growth generally emerges from gradual processes. The data indicate that simulations of dynamic increases in alpha and beta subpopulations produced more accurate results than simulations of static subpopulations. It is very likely that modeling this dynamic is dependent on cultural and spatial factors, thus making the model Taiwan-specific.

### 6.2. Impact of Drugs

**Figure 8:**
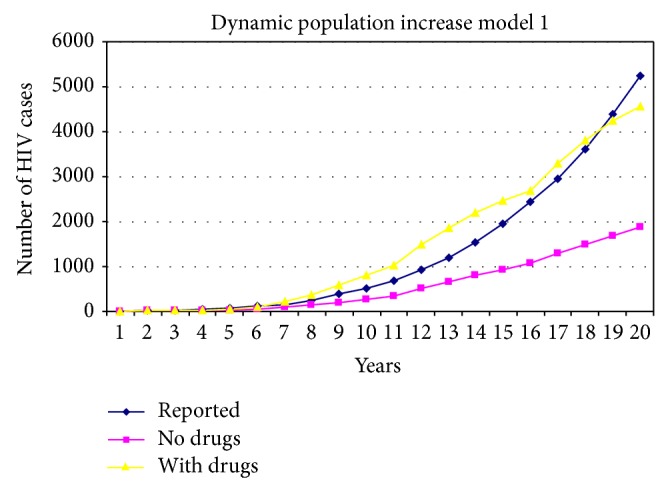
Comparison of model 1 based dynamic population increases with and without drug usage.

TCDC-sponsored research conducted by Lai [41] and Lai et al. [42] confirms that nonmedicinal drug usage exerts a major impact on the spread of HIV in Taiwan. As stated earlier, we did not address hypodermic needle sharing behavior (which occurs among both heterosexuals and homosexuals) in the present study. A secondary impact can be traced to carelessness in practicing safe sex while being under the influence of drugs [40].  Figure 8 presents the results of two simulations, one considering drug usage and one not. Both simulations assume the same sharp increases in alpha and beta populations as stated in Figure 7, model 1. The results indicate significant effects from drug usage, especially in the alpha and beta subpopulations.

### 6.3. Simulation Results Discussion

**Figure 9:**
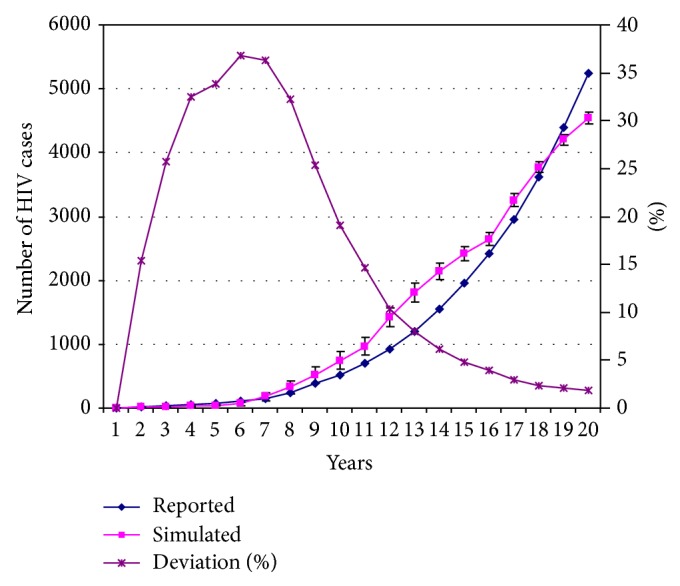
HIV infection simulation data (100 runs), including standard deviation at each point (left axis, “simulated”) and relative to the value of each point (right axis, “deviation” percentage).

Certain simulation parameters (e.g., link distribution) were implemented as random variables. The basic time unit was one month, with results reported for each year. Results from a simulation with dynamic increases in alpha and beta subpopulations, drug usage, and HIV testing are presented in Figure 9. We executed 100 runs of each simulation in an effort to reduce statistical bias. Each point along simulated curves in the figure represents the standard deviation over 100 runs; error bars represent upper and lower point values. Each point on the deviation curve represents the standard deviation over 100 runs for each point pegged to 100% of the current point value, meaning that the curve represents the relative error percentage at each point in relationship to the graph's right axis. Relative deviation from upper and lower values obtained over 100 runs was higher when the number of infected individuals was smaller, though it never exceeded 37% of the current point value. In terms of number of cases, the deviation between upper and lower values did not exceed 147 cases over an average of 1,421 for the 12th year. This is approximately 10% of the overall deviation, indicating a higher value of 1,495 infected cases and a lower value of 1,347. We also observed a connection between higher numbers of infected cases and lower dispersion levels. Combined, these results suggest that the simulations attained acceptable levels of validity in terms of reproducibility (since dispersion was limited) and credibility (since deviations across multiple runs did not affect global trends based on different policies).

**Figure 10:**
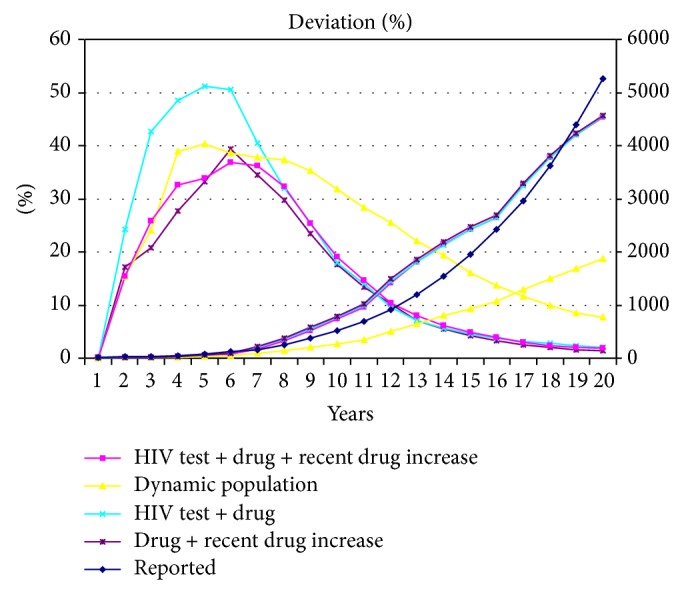
Simulation results (right axis) and percentage of relative standard deviation (left axis) for different policies. Dynamic population increases are based on model 1.

Figure 10 presents simulation and deviation results for different policies with dynamic increases in alpha and beta populations using model 1. The patterns of the deviation curve shapes and values are similar to those for actual cases. Accordingly, it is possible to provide good estimates from the simulation results across a range of accuracy values.

## 7. Conclusion

The original study goal was to establish a foundation for constructing a model capable of predicting the spread of HIV among homosexual males living in two cities in northern Taiwan. However, the project evolved into a more limited effort to create a reliable model for an agent-based simulation of a sexual activity network. It is assumed that an approach that focuses on individual agent behavior offers a new path for STI simulations. Furthermore, the results indicate that implementing rules derived from social survey statistics can, with some adjustment, accurately reflect global population behaviors that are observable in the real world. In other words, simulations in which specific behaviors are addressed on an individual level can reflect global observations without introducing unreasonably large numbers of societal rules. Such results would reflect the small-world properties inherent to scale-free networks. In the proposed model, the only rules applied at a societal level were increases in alpha and beta populations and increased drug usage. These rules were minimized to reflect global trends associated with cultural and individual tendencies. It is impossible to implement such behaviors on an agent level when a study population is as specific and limited as male homosexuals in two Taiwanese cities.

The model can be modified and improved using new data from field studies. It would be especially interesting to keep track of changes in Taiwan over time, since acknowledgment and limited acceptance of homosexuality represent a significant shift in societal attitudes. Clustering results by generation may provide insights into new behavioral trends as they emerge and improve our understanding of the effects of such factors as drug and condom usage. The biggest challenge may be organizing and making a commitment to a research project that will require several decades to complete.

## Figures and Tables

**Table 1:** Simulation parameters for our proposed agent-based HIV epidemic model.

Attribute	Data type	Description
Population size	Integer	Total number of agents.
Alpha pop. size	Integer	
Beta pop. size	Integer	
Gamma pop. size	Integer	
Max contacts	Integer	Maximum number of sexual contacts for one agent in one month.
*p*	Real	
*q*	Real	
NC	Integer	Number of sexual contacts for one agent in one month.
Delta	Integer	Number of sexual contacts with fixed partners.
Epsilon	Integer	Number of sexual contacts with nonfixed partners.

**Table 2:** Agent attributes.

Attribute	Data type	Description
ID	Integer	Serial number identifying agent in model.
State	Symbol	Epidemiological progress status.
Timer	Integer	Number of months in each epidemiological progress state.
Type	Symbol	Subpopulation type.
Fixed partners	Integer	Number of an agent's fixed partners.
Free partners	Integer	Number of an agent's nonfixed partners.
Fixed links	Integer array	IDs of fixed partners.
Free links	Integer array	IDs of nonfixed partners.
Condom usage rate	Real	1 = always use condom and 0 = never use condom.
Drug usage rate	Real	Rate of drug use.

**Table 3:** Properties and methods for each simulation component.

Component	Properties	Methods
Observer	Higher in class hierarchy.	Uses information from classes lower in the hierarchy to create screen display.

World	Two-dimensional lattice containing agents.	Defines social network structure. Can be influenced by agent behavior.

Scheduler	Defines time in one-month units.	Defines events that happen to each agent during each time unit and ensures that rules are followed.

Rules	Rules applied to simulation pertaining to contamination, HIV evolution, epidemiological progress status, and so forth.	Communicates rules to schedulers and agents.

Agent	One agent represents one human individual.	Changes status according to epidemic model parameters, capable of interacting with other agents.

Agent behavior	Behavioral rules based on statistical data from surveys.	Controls agent behaviors (e.g., number of contacts, condom usage).

## References

[1] World Health Organization (2009). *AIDS Epidemic Update*.

[2] Taiwan Centers for Disease Control (TCDC) (2009). *Statistics of Communicable Diseases and Surveillance Report, Republic of China, 2008*.

[3] Jia Y., Aliyu M. H., Huang Z. J. (2014). Dynamics of the HIV epidemic in MSM.

[4] Li X., Lu H., Cox C. (2014). Changing the landscape of the HIV epidemic among MSM in China: results from three consecutive respondent-driven sampling surveys from 2009 to 2011. *BioMed Research International*.

[5] Watts D. J., Strogatz S. H. (1998). Collective dynamics of 'small-world' networks. *Nature*.

[6] Albert R., Barabási A.-L. (2002). Statistical mechanics of complex networks. *Reviews of Modern Physics*.

[7] Barabási A.-L., Albert R. (1999). Emergence of scaling in random networks. *Science*.

[8] Newman M. E. J. (2000). Models of the small world: a review. *Journal of Statistical Physics*.

[9] Newman M. E. J., Watts D. J. (1999). Scaling and percolation in the small-world network model. *Physical Review E*.

[10] Brailsford S. C., Shahani A. K., Roy R. B., Sivapalan S. (1992). Simulation modelling for HIV infection and AIDS. *International Journal of Bio-Medical Computing*.

[11] Paltiel A. D., Scharfstein J. A., Seage G. R. (1998). A Monte Carlo simulation of advanced HIV disease: application to prevention of CMV infection. *Medical Decision Making*.

[12] Peterson D., Willard K., Altmann M., Gatewood L., Davidson G. (1990). Monte Carlo simulation of HIV infection in an intravenous drug user community. *Journal of Acquired Immune Deficiency Syndromes*.

[13] Bearman P. S., Moody J., Stovel K. (2004). Chains of affection: the structure of adolescent romantic and sexual networks. *American Journal of Sociology*.

[14] Jones J. H., Handcock M. S. (2003). An assessment of preferential attachment as a mechanism for human sexual network formation. *Proceedings of the Royal Society B: Biological Sciences*.

[15] Jones J. H., Handcock M. S. (2003). Sexual contacts and epidemic thresholds. *Nature*.

[16] Klovdahl A. S., Potterat J. J., Woodhouse D. E., Muth J. B., Muth S. Q., Darrow W. W. (1994). Social networks and infectious disease: the Colorado Springs Study. *Social Science & Medicine*.

[17] Kretzschmar M. (2000). Sexual network structure and sexually transmitted disease prevention: a modeling perspective. *Sexually Transmitted Diseases*.

[18] Morris M. (1997). Sexual networks and HIV. *AIDS*.

[19] Potterat J. J., Phillips-Plummer L., Muth S. Q. (2002). Risk network structure in the early epidemic phase of HIV transmission in Colorado Springs. *Sexually Transmitted Infections*.

[20] Benkimoun P. (2004). Quarante millions de personnes vivent avec le virus du sida. *Le Monde*.

[21] Liljeros F., Edling C. R., Amaral L. A. N., Stanley H. E., Åberg Y. (2001). Social networks: the web of human sexual contacts. *Nature*.

[22] Liljeros F., Edling C. R., Amaral L. A. N. (2003). Sexual networks: implications for the transmission of sexually transmitted infections. *Microbes and Infection*.

[23] Liljeros F., Edling C. R., Stanley H. E., Aberg Y., Amaral L. A. (2003). Distributions of number of sexual partnerships have power-law decaying tails and finite variance. *Working Paper*.

[24] Schneeberger A., Mercer C. H., Gregson S. A. J. (2004). Scale-free networks and sexually transmitted diseases: a description of observed patterns of sexual contacts in Britain and Zimbabwe. *Sexually Transmitted Diseases*.

[25] Teweldemedhin E., Marwala T., Mueller C. Agent-based modelling: a case study in HIV epidemic.

[26] Zvirinsky P., Laansky J. A bottom-up approach to immune system modelling.

[27] Huang C. Y., Sun C. T., Hsieh J. L., Lin H. (2004). Simulating SARS: small-world epidemiological modeling and public health policy assessments. *Journal of Artificial Societies and Social Simulation*.

[28] Huang C.-Y., Sun C.-T., Lin H.-C. (2005). Influence of local information on social simulations in small-world network models. *Journal of Artificial Societies and Social Simulation*.

[29] Xiaoguang G., Zhicheng L. (2003). Study on multi-agent simulation of new product market diffusion. *System Engineering Theory and Practice*.

[30] Zhou H. (2005). Research of the small-world character during Rumor's propagation. *Journal of Wuhan University of Science and Engineering*.

[31] Watts D. J. (2003). *Six Degrees: The Science of a Connected Age*.

[32] Kermack W. O., McKendrick A. G. (1927). Contributions to the mathematical theory of epidemics. *Proceedings of the Royal Society London*.

[33] Catania J. A., Coates J. T., Kegels S., Fullilove T. M. (1992). The population-based AMEN (AIDS in multi-ethnic neighborhoods) study. *The American Journal of Public Health*.

[34] Morris M., Mollison D. (1995). Data driven network models for the spread of infectious disease. *Epidemic Models: Their Structure and Relation to Data*.

[35] Shiboski S., Padian N. S. (1996). Population- and individual-based approaches to the design and analysis of epidemiologic studies of sexually transmitted disease transmission. *The Journal of Infectious Diseases*.

[36] Haraldsdottir S., Gupta S., Anderson R. M. (1992). Preliminary studies of sexual networks in a male homosexual community in Iceland. *Journal of Acquired Immune Deficiency Syndromes*.

[37] Wylie J. L., Jolly A. (2001). Patterns of chlamydia and gonorrhea infection in sexual networks in Manitoba, Canada. *Sexually Transmitted Diseases*.

[38] Marsden P. V. (1990). Network data and measurement. *Annual Review of Sociology*.

[39] Amaral L. A. N., Scala A., Barthélémy M., Stanley H. E. (2000). Classes of small-world networks. *Proceedings of the National Academy of Sciences of the United States of America*.

[40] Chen M. A., Lai S. F., Lan Y. C., Chen K. H., Chen Y. J. Molecular epidemiology of HIV-1 infection among injecting drug users in Taiwan-report of an emergent situation.

[41] Lai S. F. (2003). *Molecular epidemiology of HIV-1 infection in men who have sex with men from gay Saunas in Taiwan [Ph.D. Dissertation]*.

[42] Lai S. F., Hong C. P., Lan Y. C. Molecular epidemiology of HIV-1 in men who have sex with men from gay Saunas in Taiwan from 2000 to 2003.

[43] Yamamoto T., Satoko I. Fighting a rising tide: the response to AIDS in East Asia.

